# Upregulation of long non-coding RNA PlncRNA-1 promotes proliferation and induces epithelial-mesenchymal transition in prostate cancer

**DOI:** 10.18632/oncotarget.15318

**Published:** 2017-02-13

**Authors:** Yang Jin, Zilian Cui, Xudong Li, Xunbo Jin, Jian Peng

**Affiliations:** ^1^ Department of Hepatobiliary Surgery, Xiangya Hospital, Central South University, Changsha, Hunan, China; ^2^ Shandong University School of Medicine, Jinan, Shandong, China; ^3^ Minimally Invasive Urology Center, Shandong Provincial Hospital affiliated to Shandong University, Jinan, Shandong, China; ^4^ Department of Urology, Binzhou People's Hospital, Binzhou, Shandong, China

**Keywords:** PlncRNA-1, long noncoding RNA, LNCAP, C4-2, TGF-β1

## Abstract

**Objective:**

To confirm that PlncRNA-1 regulates the cell cycle in prostate cancer cells and induces epithelial-mesenchymal transition (EMT) in prostate cancer through the TGF-β1 pathway.

**Results:**

PlncRNA-1 and TGF-β1 expression levels were significantly higher in prostate cancer tissues than in normal prostate tissues (*P* < 0.05) and were significantly positively correlated. TGF-β1, N-cadherin and Cyclin-D1 were downregulated and E-Cadherin was upregulated in LNCAP cells after silencing of PlncRNA-1, as determined by real-time PCR and Western blot. TGF-β1, N-cadherin and Cyclin-D1 were upregulated and E-cadherin was downregulated in C4-2 cells, as determined by real-time PCR and Western blot. Overexpression of PlncRNA-1 in C4-2 cells was observed when TGF-β1 inhibitor LY2109761 was added. Western blot analysis showed that compared with their expression when TGF-β1 inhibitor LY2109761 was not added, N-Cadherin and CyclinD1 expression decreased and E-Cadherin expression increased. Transwell results showed that the invasive ability of C4-2 cells was enhanced after overexpression of PlncRNA-1, and the invasion ability was decreased after addition of TGF-β1 inhibitor LY2109761. The cell cycle was blocked by overexpression of PlncRNA-1 in C4-2 and by the addition of TGF-β1 inhibitor LY2109761, as determined by flow cytometry. In vitro experiments showed that PlncRNA-1 can regulate the growth of prostate cancer cells and EMT through the TGF-β1 pathway. *In vivo* experiments also confirmed the above results. Tumor growth was significantly blocked by overexpressing PlncRNA-1 in C4-2 cells and by the TGF-β1 inhibitor LY2109761 in animal experiments.

**Materials and Methods:**

The expression levels of PlncRNA-1 and TGF-β1 were analyzed in 19 prostate cancer tissue samples and in adjacent normal tissue samples, 4 Pca cell lines, including LNCaP, C4-2, DU145, and PC3, and 1 normal prostate epithelial cell line RWPE-1. LNCAP cells were divided into the LNCAP control group and the LNCAP-PlncRNA-1-siRNA group. Cells from the prostate cancer cell line C4-2 were divided into the C4-2 control group and the C4-2-PlncRNA-1 experimental group. Changes in TGF-β1, E-cadherin and N-cadherin were detected by qPCR and Western Blot assay after silencing and overexpression of PlncRNA-1. The cell cycle, cell invasion, and levels of Cyclin-D1, E-Cadherin, and N-Cadherin were observed after adding TGF-β1 inhibitor LY2109761 in the C4-2-PlncRNA-1 group. The effects of TGF-β1 inhibitor LY2109761 on the tumorigenicity of C4-2 cells after overexpression of PlncRNA-1 was investigated *in vivo*.

**Conclusions:**

PlncRNA-1 is an oncogene that regulates the cell cycle, cyclin-D1 and EMT in prostate cancer cells through the TGF-β1 pathway.

## INTRODUCTION

Prostate cancer (PCa) is one of the most common malignant tumors of the male genitourinary system. In 2016, the American Cancer Society reported an estimated 180,890 new prostate cancer cases, accounting for 21% of cancers in men and making PCa the most prevalent cancer among males. PCa also exhibits the second highest mortality rate among cancers, with an estimated 26,120 PCa-attributed deaths [1].

We found that PlncRNA-1 plays an important role in the pathogenesis of PCa. In addition to reciprocally regulating the androgen receptor (AR), PlncRNA-1 knockdown induces tumor cell apoptosis [2]. In the present study, we found that PlncRNA-1 regulates the expression of TGF-β1 during apoptosis. Thus, we propose that PlncRNA-1-induced apoptosis may act through the TGF-β1 signaling pathway.

Transforming growth factor-β (TGF-β1) is a family of proteins that regulate cell growth and differentiation. TGF-β1 can transform the normal fibroblast phenotype. As an epidermal growth factor, TGF-β1 changes fibroblast growth characteristics, including loss of adherence, growth capacity and growth capacity in agar [3].

TGF-β1 can exert two opposite effects on tumors: in the early stages of tumor development, it can act as a tumor suppressor to suppress tumor growth because of its role in blocking the growth cycle. During tumor progression, TGF-β1 can be produced by tumor cells and/or the surrounding stromal cells; the growth inhibition effect of TGF-β1 is reduced or eliminated, and thus, tumor cell growth becomes dominant. In the late stages of tumor growth, TGF-β1 acts as a tumor promoting factor by stimulating angiogenesis, promoting cell proliferation, inhibiting immune cells and inducing synthesis of the extracellular matrix to promote tumor growth, invasion and metastasis [4]. A number of studies have shown that liver, gastric, colon, prostate, breast, lung and esophageal cancers have high levels of TGF-β1 expression, and many tumor cell lines can secrete TGF-β1 [5–11].

Clinically, inhibition of TGF-β1 can prevent the metastasis of tumor cells and can also change the surrounding environment, including vascular regeneration, matrix activation and immunosuppression [12]. Inhibition of the TGF-β1 signaling pathway may provide a basis for the treatment of cancer.

PlncRNA-1 is a long noncoding RNA that was first identified in prostate cancer, but its role in the development and progression of prostate cancer has not been completely elucidated [2]. Here, we show that PlncRNA-1 and TGF-β1 expression are highly correlated. Downregulation of PlncRNA-1 induced apoptosis and caused a decrease of TGF-β1. This discovery provides a theoretical basis for the prevention and treatment of and for the development of interventions for prostate cancer.

## RESULTS

### Overexpression of PlncRNA-1 and TGF-β1 in prostate cancer cells and tissues

**Figure 1:**
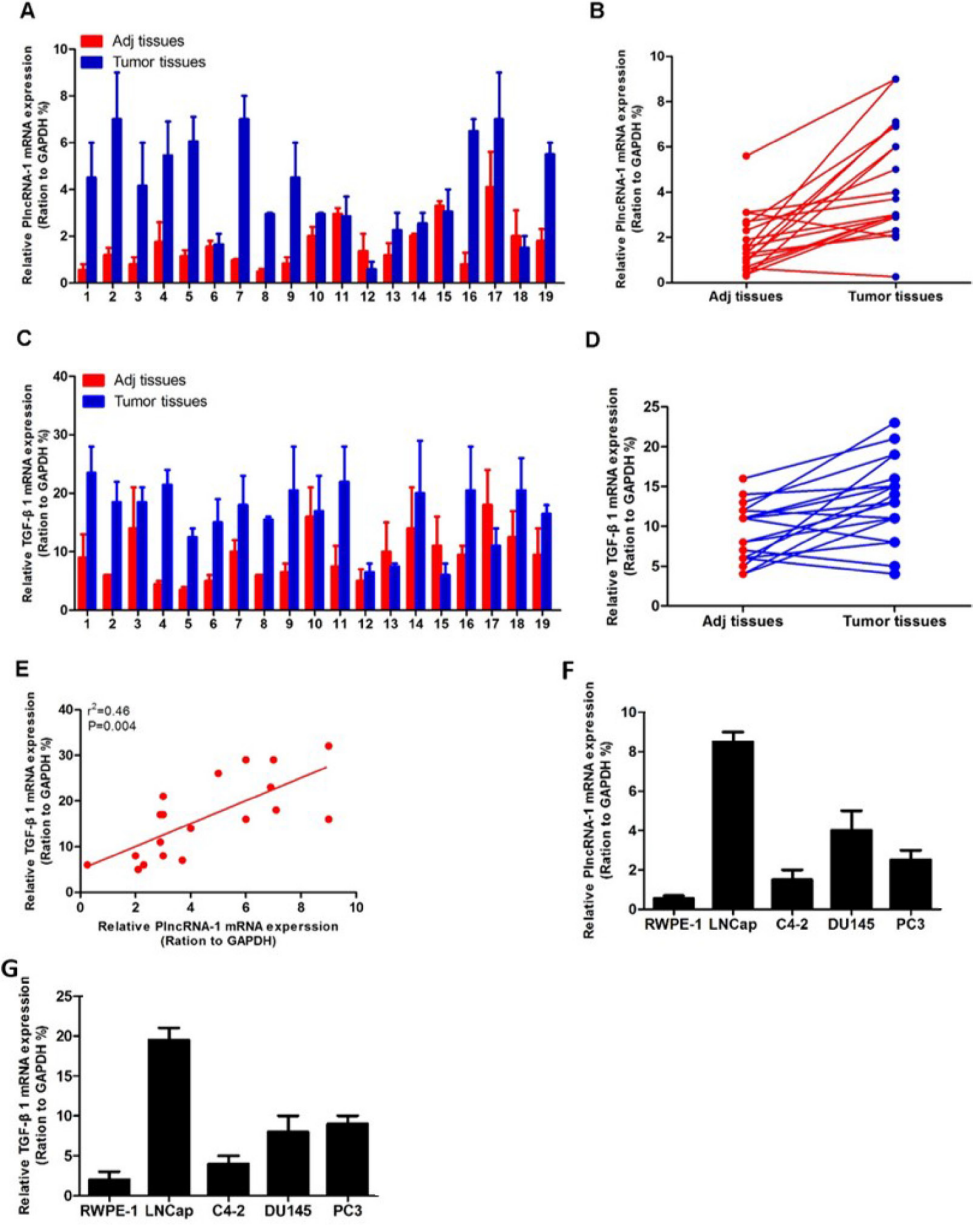
Expression of PlncRNA-1 and TGF-β1 in prostate cancer and correlation analysis (**A**, **B**) Quantitative RT-PCR analysis of PlncRNA-1 expression in prostate cancer and adjacent normal tissue. (**C**, **D**) Quantitative RT-PCR analysis of TGF-β1 expression in prostate cancer and adjacent normal tissue. (**E**) There is a high correlation between PlncRNA-1 and TGF-β1 expression. (**F**, **G**) PlncRNA-1 and TGF-β1 expression levels are determined by qPCR in 5 prostate cell lines.

We detected TGF-β1 and PlncRNA-1 expression in 19 cases of PCa using real-time PCR. The high expression of PlncRNA-1 in PCa tissues was consistent with our previous findings. TGF-β1 was also highly expressed in PCa tissues (Figure 1A–1D). Based on a linear correlation analysis (r^2^ = 0.46), the expression levels of these genes were highly correlated, and the differences between the conditions were significant (*P* = 0.004; Figure 1E). The expression of PlncRNA-1 and TGF-β1 were higher in 4 cancer cell lines including LNCaP, DU145, PC3, and C4-2 compared with 1 normal prostate epithelial cell line, RWPE-1 (Figure 1F, 1G). Some researchers have found that TGF-β1 is closely related to angiogenesis, metastasis and prognosis of prostate cancer. Preoperative expression levels can predict the progression of prostate cancer after radical prostatectomy [13, 14], suggesting that TGF-β1 plays an important role in the development of PCa.

### Knockdown of PlncRNA-1 decreases TGF-β1 in LNCaP cells, and overexpression of PlncRNA-1 upregulates TGF-β1 in C4-2 cells

**Figure 2:**
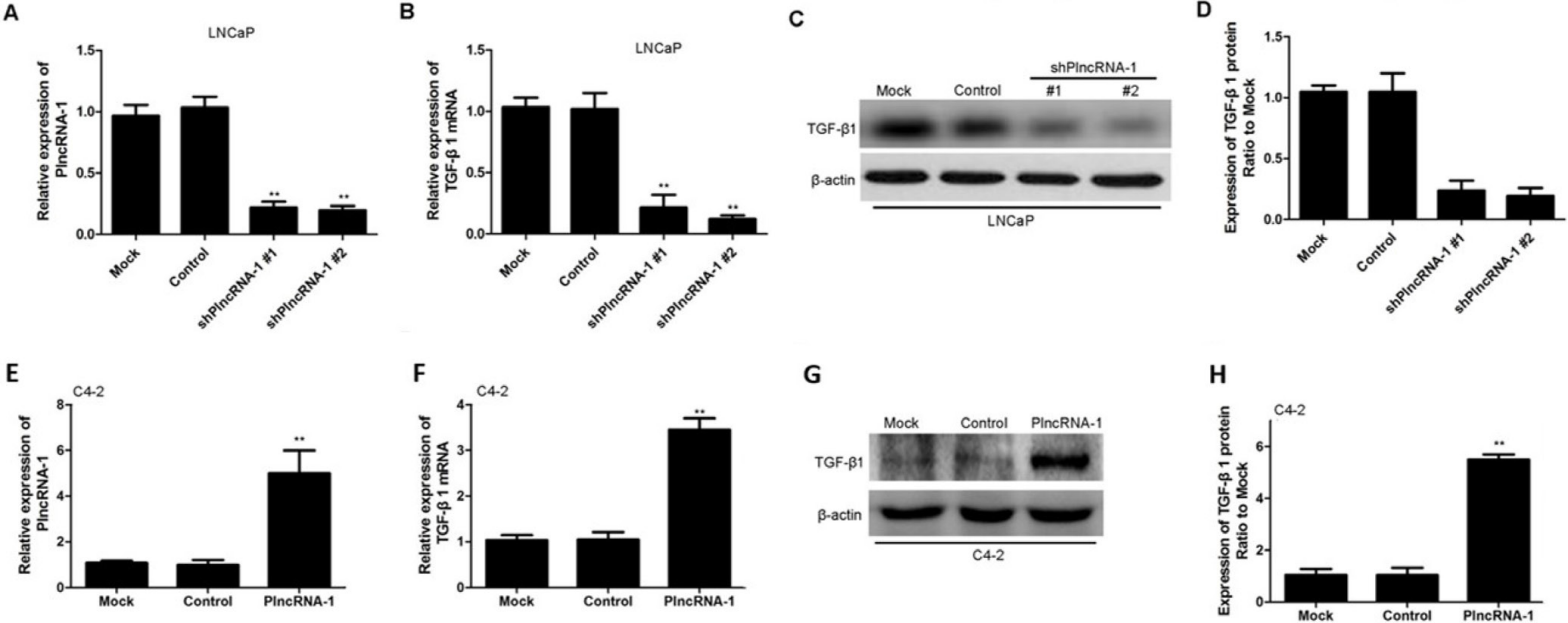
TGF-β1 expression in LNCaP and C4-2 cells after silencing and overexpression of PlncRNA-1 (**A**, **B**) Knockdown of PlncRNA-1 decreased TGF-β1 expression, as assessed by qPCR in LNCaP cells. (**C**, **D**) Knockdown of PlncRNA-1 decreased TGF-β1 expression, as assessed by Western blot in LNCaP cells. (**E**, **F**) Overexpression of PlncRNA-1 upregulated TGF-β1, as assessed by qPCR in C4-2 cells. (**G**, **H**) Overexpression of PlncRNA-1 upregulated TGF-β1 expression, as assessed by Western blot in C4-2 cells.

We synthesized two shPlncRNA-1 constructs to silence PlncRNA-1 and transfected them into LNCaP cells. Quantitative RT-PCR analysis showed that compared with the levels in the mock control group, PlncRNA-1 levels were significantly lower in the treatment group, indicating that both shPlncRNA-1 constructs produced obvious interference effects (Figure 2A). Based on qRT-PCR and Western blot analysis, TGF-β1 mRNA and protein levels in the treatment group were significantly decreased compared with those in the control groups (Figure 2B–2D). Following overexpression of PlncRNA-1 in C4-2 cells, TGF-β1 levels were significantly higher in the treatment group than in the mock and control groups, as assessed by qRT-PCR and Western blot (Figure 2E–2H). These results suggest that the regulation of PlncRNA-1 expression may significantly affect the TGF-β1 signaling pathway and that downregulating PlncRNA-1 should reduce TGF-β1 expression and improve tumor prognosis.

### Effect of PlncRNA-1 on the epithelial-mesenchymal transition (EMT) in prostate cancer cells

**Figure 3:**
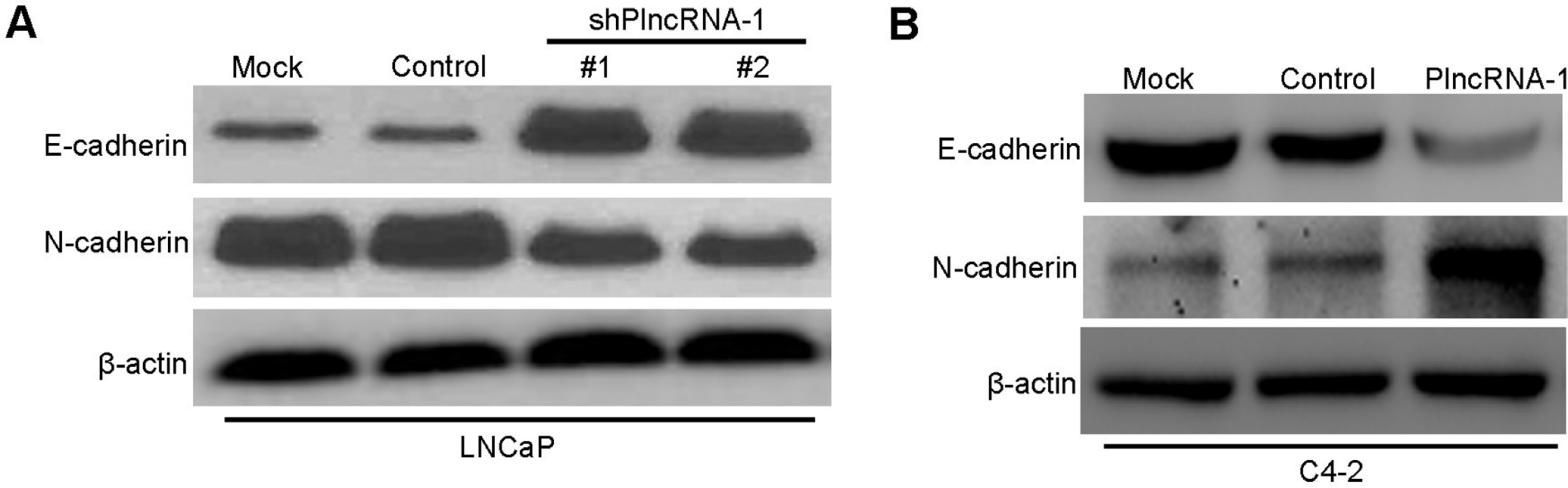
Effect of PlncRNA-1 on EMT in LNCaP and C4-2 cells after silencing and overexpression of PlncRNA-1 (**A**) Western Blot shows that knockdown of PlncRNA-1 decreased N-Cadherin and upregulated E-Cadherin in LNCaP cells. (**B**) Western Blot shows that overexpression of PlncRNA-1 upregulated N-Cadherin and decreased E-Cadherin in C4-2 cells.

We synthesized two shPlncRNA-1 constructs to silence PlncRNA-1 and transfected them into LNCaP cells. Western blot analysis shows that compared with the mock and control group, N-Cadherin levels decreased and E-cadherin levels increased in the treated group (Figure 3A). Upon overexpression of PlncRNA-1 in C4-2 cells, the expression of N-Cadherin increased and the expression of E-cadherin decreased in the treated group (Figure 3B). These results suggest that the expression of PlncRNA-1 is related to EMT in prostate cancer.

### LY2109761, a TGF-β1 inhibitor, blocked EMT caused by PlncRNA-1 in C4-2 cells

**Figure 4:**
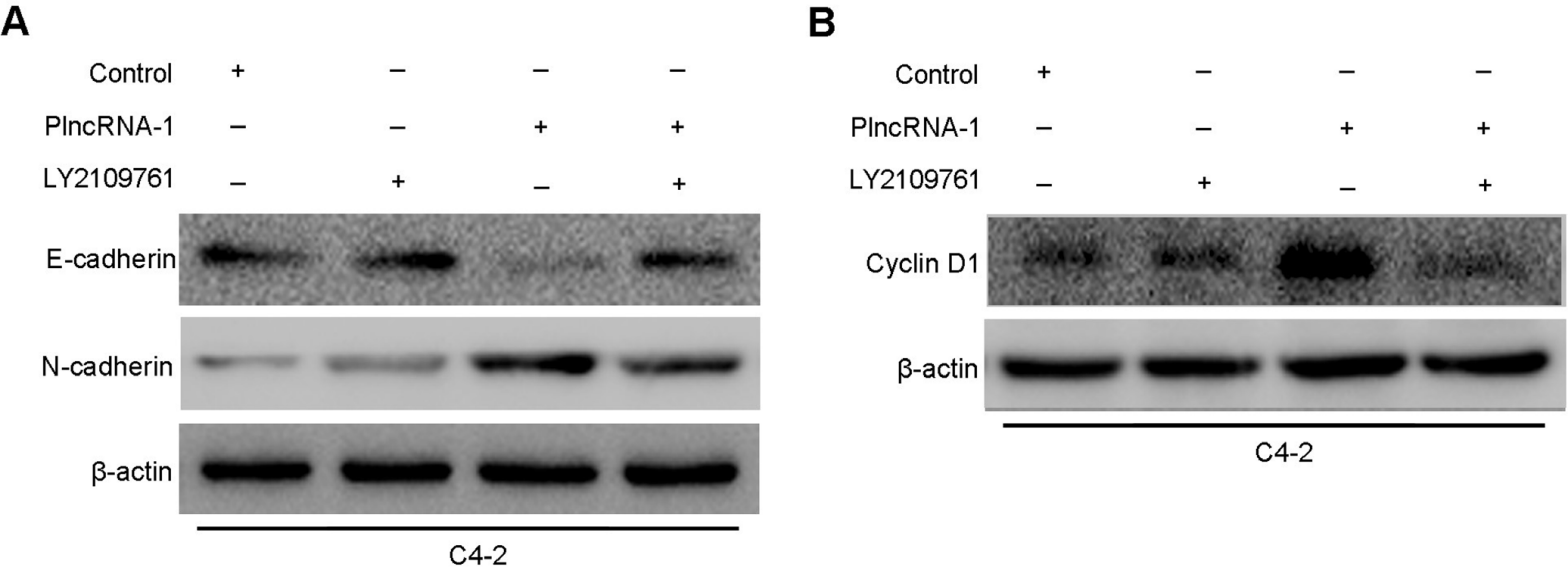
Effects of PlncRNA-1 overexpression and TGF-β1 inhibitor LY2109761 addition on EMT and CyclinD1 in C4-2 cells (**A**) Effect of PlncRNA-1 overexpression on EMT in C4-2 cells after the addition of TGF-β1 inhibitor LY2109761. (**B**) Effect of PlncRNA-1 overexpression on CyclinD1 in C4-2 cells after the addition of TGF-β1 inhibitor LY2109761.

Overexpression of PlncRNA-1 in C4-2 was observed after adding TGF-β1 inhibitor LY2109761. Western blotting analysis showed that compared with their expression without the addition of TGF-β1 inhibitor LY2109761, the expression of N-Cadherin decreased and the expression of E-cadherin increased (Figure 4A), while the expression of CyclinD1 decreased (Figure 4B). Compared with the effect in the control cells, the effect of the overexpression of PlncRNA-1 in C4-2 cells disappeared (Figure 4A, 4B), indicating that PlncRNA-1 regulates the prostate cancer cell cycle and EMT through TGF-β1 pathway.

### LY2109761 blocks the effect of PlncRNA-1 on the cell cycle and invasion in C4-2 cells

**Figure 5:**
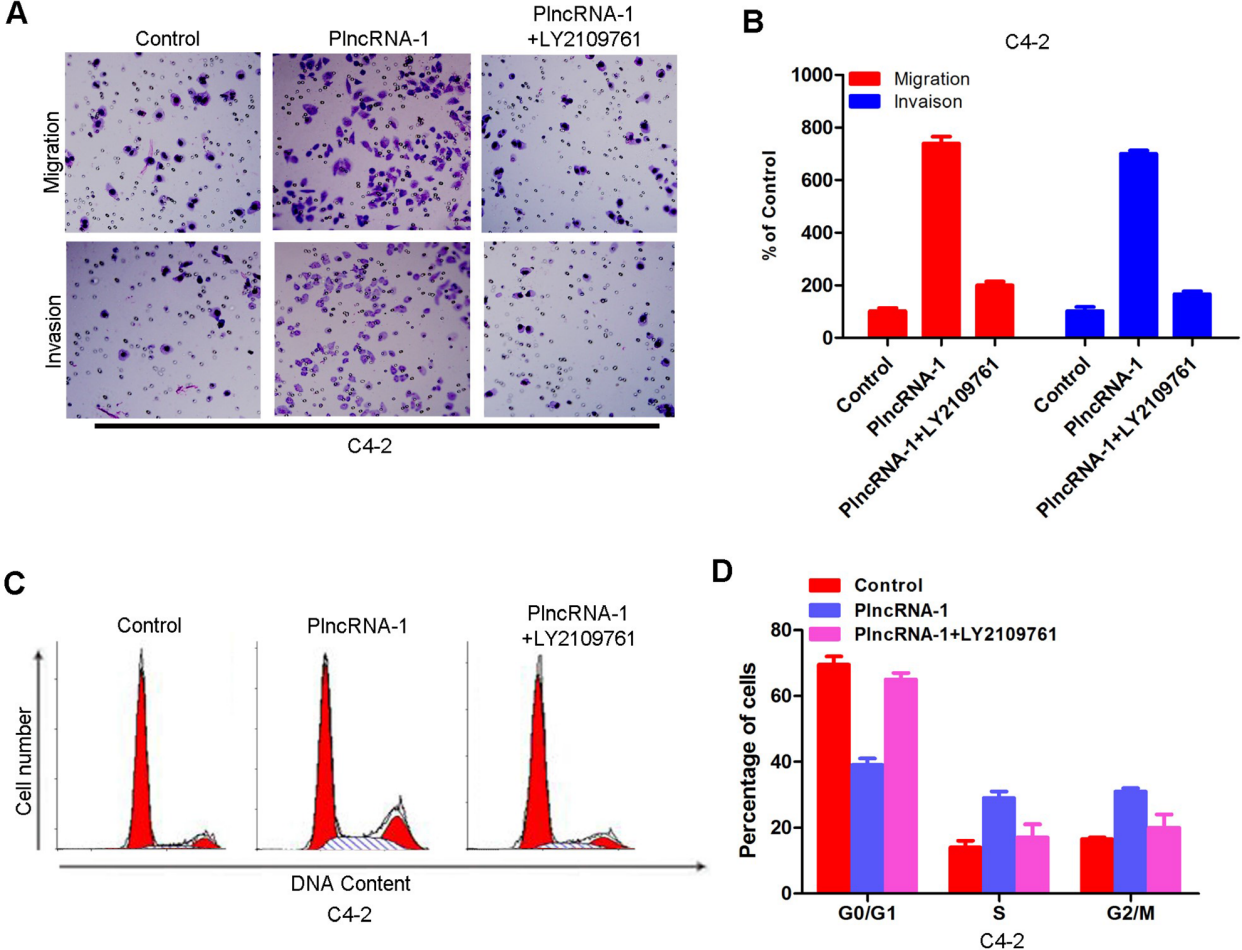
Effects of PlncRNA-1 overexpression and the addition of TGF-β1 inhibitor LY2109761 on invasion and the cell cycle in C4-2 cells (**A**, **B**) Transwell analysis was performed on C4-2 cells after the addition of TGF-β1 inhibitor LY2109761 and overexpression of PlncRNA-1. (**C**, **D**) Fluorescence activated cell sorting (FACS) analysis was performed on C4-2 cells after the addition of TGF-β1 inhibitor LY2109761 and overexpression of PlncRNA-1.

After overexpression of PlncRNA-1 and addition of TGF-β1 inhibitor LY2109761, transwell detection showed that increased invasion ability of C4-2 cells was blocked (Figure 5A, 5B). These results suggest that PlncRNA-1 affects the invasive ability of cells through the TGF-β1 pathway. The effect of PlncRNA-1 on the cell cycle was blocked by the addition of TGF-β1 inhibitor LY2109761, as shown by flow cytometry (Figure 5C, 5D).

### Tumor growth in nude mice following overexpression of PlncRNA-1 and addition of TGF-β1 inhibitor LY2109761 in C4-2 cells

**Figure 6:**
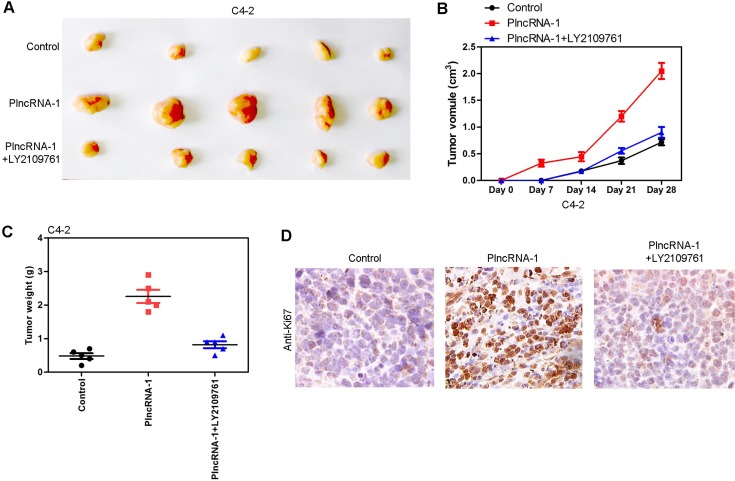
Tumor growth in nude mice following overexpression of PlncRNA-1 and the addition of TGF-β1 inhibitor LY2109761 in C4-2 cells (**A**, **C**) Compared with the control group, the tumorigenic ability of C4-2 cells was enhanced after overexpression of PlncRNA-1, and tumor forming ability decreased after adding TGF-β1 inhibitor LY2109761. (**B**) After subcutaneous implantation of tumor cells, the growth rate of C4-2 was significantly increased after overexpression of PlncRNA-1, and the growth rate was decreased after adding TGF-β1 inhibitor LY2109761. (**D**) Ki-67 expression was detected by immunohistochemistry.

In animal experiments, 15 nude mice were divided into an C4-2 control group (5 mice), an C4-2-PlncRNA-1 group (5 mice) and an C4-2-PlncRNA-1 + LY2109761 group. Tumor cells were implanted subcutaneously in nude mice. Tumor volume was measured every week. After 4 weeks, the tumors were weighed and measured. The results show that the growth rate of the C4-2-PlncRNA-1 group was faster than that of the C4-2 control group, and the tumor weight was higher than that of the C4-2 group, which was consistent with previous research findings (Figure 6A, 6C). After the addition of TGF-β1 inhibitor LY2109761, tumor growth was significantly blocked (Figure 6B). The expression of Ki-67 was also decreased by immunohistochemistry (Figure 6D). The results demonstrate that PlncRNA-1 can affect the growth of tumor cells *in vivo* through the TGF-β1 pathway.

## DISCUSSION

In Europe and other developed countries, PCa is one of the most common and malignant cancers [1]. When PCa is at the early stages, a variety of treatment options are available, and the disease can be cured by surgery or radiotherapy. However, if PCa has advanced to the castration-resistant stage, there is no effective treatment, and care is primarily palliative. To investigate the pathogenesis of PCa, we applied next-generation sequencing techniques to screen for abnormal gene expression levels in prostate carcinoma samples relative to adjacent tissues. Our results showed that PlncRNA-1 was upregulated to very high levels in cancerous tissues [2, 15]. In our study, we confirmed that PlncRNA-1 acts as an oncogene in prostate cancer, consistent with our previous findings. By modulating PlncRNA-1 levels, we found that changes in TGF-β1, cyclin D1, N-Cadherin and E-Cadherin are highly correlated with changes in PlncRNA-1.

The tumor cell load was related to the high level of TGF-β1 in the plasma and the excretion of TGF-β1 in urine. The relationship between TGF-β1 and tumors is most obvious in the advanced stage, in which the secretion and activity of TGF-β1 increases, providing a suitable microenvironment for tumor survival and progression [4]. The overexpression of TGF-β1 in tumors indicates more aggressive cancer and poor prognosis [13, 14]. By regulating PlncRNA-1, we could regulate TGF-β1 expression and induced changes in the cell cycle, Cyclin-D1 and EMT. Thus, we hypothesized that we could regulate EMT by regulation of PlncRNA-1 at the gene level in prostate cancers to improve the prognosis of prostate cancer patients and provide new interventions for their treatment.

LncRNAs, derived from genes, enhancer elements, introns, antisense strands of the gene, or other elements of the genome, are a class of RNAs that include RNAs over 200 nucleotides in length [16, 17]. LncRNAs possess primary sequence information, which can interact with nucleic acid molecules through base-pair complementarity. Meanwhile, lncRNAs can interact with proteins and nucleic acid molecules and even RNA-RNA proteins and RNA-DNA-protein complexes through their secondary structures [18]. LncRNAs have a range of different characteristics and a number of flexible secondary structures. There are at least four interacting partners of LncRNAs, which included signal molecules, bait, guides, and scaffolds [17, 19]. Upon further study of LncRNAs, long chain non-coding RNAs were found to be involved in the regulation of cellular activities, including imprinting, X chromosome inactivation, stem cell differentiation and tumor metastasis [16, 20].

Many lncRNAs function through binding to the PRC family protein by trans-action, and HOTAIR was the first non-coding gene discovered in this way [21]. The role of HOTAIR in breast cancer was reported in 2010 [22]. The expression level of lncRNAs were positively correlated with the degree of tumor cell malignancy [22]. The expression level of HOTAIR that has a clear correlation with prognosis. The lncRNAs is located at the HOXC site of chromosome 12, knocking out the lncRNA does not affect the gene expression at that locus, and the gene at the HOXD locus of chromosome 2 was significantly upregulated, indicating that the lncRNA regulated gene expression by trans-action [23].

In addition to HOTAIR, some lncRNAs can also bind directly to transcription factors. In 2014, Yang reported on the lncRNA BCAR4 (breast-cancer anti-estrogen resistance 4), which simultaneously binds to the transcription factor SNIP1 (Smad nuclear interacting protein 1) and the phosphatase 1 nuclear- Targeting subunit (PNUTS), and it regulates the hedgehog/GLI2 signaling pathway [24].

The function of some lncRNAs are also related to variable shear of mRNA except in transcriptional regulation. LncRNA ZEB2AS1 can promote EMT by regulating the variable shear, which promotes tumor metastasis. In addition to ZEB2-AS1, MALAT1 also regulates variable shear. MALAT1, located in the nucleus, can regulate the precursor RNA splicing protein SR and control its location and activity. In this way, MALAT1 regulates the variable shear [25, 26].

The role of lncRNAs is not limited to the nucleus; they also function in the cytoplasm. Some lncRNAs play the role of a sponge by binding miRNAs, thereby regulating the miRNA binding target gene. LncRNA-ATB is an lncRNA located downstream of TGF-β1 [27]. TGF-β1 signaling pathways play an important role in development and homeostasis, but once the cells become tumorigenic, they can use the TGF-β1 signaling pathway to promote EMT characteristics to obtain migration ability [28, 29]. The miR-200 family regulates these two genes by binding to the 3′UTR region of ZEB1/2, which regulates EMT [30, 31]. LncRNA-ATB contains three miR-200 binding sites that competitively bind miR-200, thereby upregulating the RNA level of the ZEB1/2 gene and inducing EMT. LncRNA-ATB not only competitively binds miR-200 as a sponge but also binds to IL-11 mRNA. This observation indicates the flexibility of lncRNAs in their mechanism of action and their time-specific effect and shows that lncRNAs themselves are subject to fine regulation.

The PlncRNA-1 RNA also acts as a sponge to protect the AR from inhibition by miR-34c and miR-297 in prostate cancer. This is one of the mechanisms that play a role in prostate cancer [32].

In the cytoplasm, some lncRNAs interact directly with the target protein in addition to absorbing miRNAs as sponges. NKILA is a tumor suppressor gene that interacts directly with NF-κB [33]. The discovery of lncRNA indicates the complexity of the regulation of the NF-κB signaling pathway but also reveals a new mechanism of action of lncRNAs, which can interact with a target protein and affect the function of a target gene.

In conclusion, lncRNAs can play a variety of roles, but due to the mechanistic complexity, there are no comprehensive studies clarifying their functions. This study found that PlncRNA-1 acts as a cancer gene in prostate cancer, and it can regulate the cell cycle and Cyclin-D1. Additionally, PlncRNA-1 can regulate prostate cancer cell growth and prostate cancer EMT through the TGF-β1 pathway. It is not clear whether the interaction between PlncRNA-1 and TGF-β1 is direct or indirect, and this issue needs to be further studied.

## MATERIALS AND METHODS

### Samples, cells, and antibodies

Frozen PCa tissue was obtained with informed consent from patients who underwent radical resections at the Shandong Provincial Hospital. Ethical consent was granted by the Committees for Ethical Review of Research involving Human Subjects of Shandong Provincial Hospital. LNCaP, C4-2, DU145, and PC3 cells were cultured in Dulbecco's modified Eagle's medium (Invitrogen, Carlsbad, CA, USA), supplemented with 10% fetal bovine serum and kept at 37°C and 5% CO_2_. The normal prostate cell line RWPE-1 was cultured in keratinocyte serum free medium (GIBICO, 17005–042, Grand Island, NY. Monoclonal TGF-β1 (ab9758), E-cadherin(ab76055),N-cadherin (ab18203), Cyclin D1 (ab137875), and β actin (ab8226) antibodies were got from Abcam (Cambridge, MA, USA). LY2109761 (No: S2704) was purchased from Selleck (Houston, TX, USA).

### Plasmid construction and transfection

For overexpression analyses, cDNA encoding the complete open reading frame of PlncRNA-1 was cloned into the pBabe vector to generate the PlncRNA-1 expression plasmid. The expression plasmid was verified by sequencing both strands and was used to transfect C4-2 cells to establish the PlncRNA-1 overexpression cell line. For PlncRNA-1 RNA interference, control (pSuper) and pSuper-shPlncRNA-1s plasmids were purchased from Shanghai Genechem Co., LTD (Shanghai, China) and were used to transfect LNCaP cells to establish the PlncRNA-1 knockdown cell line. The transfection efficiency of PlncRNA-1 was confirmed by quantitative reverse transcription PCR (qRT-PCR) analysis.

### Western blotting

Equal amounts of protein were separated on SDS polyacrylamide gels and were electrotransferred to polyvinylidene fluoride membranes (Millipore, Bedford, MA, USA). The membranes were immunoblotted overnight at 4°C with primary antibodies followed by the corresponding secondary antibodies. β-actin was used as the loading control.

### Quantitative reverse-transcription PCR

RNA was extracted using TRIzol reagent according to the manufacturer's recommended protocol (Invitrogen). qRT-PCR was performed using the StepOne and StepOne Plus Real-Time PCR Systems (Applied Biosystems, Foster City, CA, USA). GAPDH was used as a loading control. The primers used in this study were 5′-AGT AGT TGC TTG TCC TAT-3′ and 5′-AAG TCA GTA AGT CCT AAG T-3′ for PlncRNA-1,5′-ATG GCA GCG ACC ATA CTC CTC TTT-3′ and 5′-AAA GAC AGC CAC TCA GGC GTA TCA-3′ for TGF-β1,5′-GAG CGA GAC CCC ACT AAC AT-3′ and 5′-TTC ACA CCC ATC ACA AAC AT-3′ for GAPDH. The experiments were repeated a minimum of 3 times.

### Flow cytometry analysis of the cell cycle

To assay the cell cycle stage, exponentially growing cells were trypsinized, rinsed twice with ice-cold phosphate-buffered saline (PBS), and fixed in 75% ice-cold ethanol. The fixed cells were washed with ice-cold PBS and incubated at 37°C for 30 min in 1 ml of PBS containing 20 μg ml−1 RNase A (Fermentas) and stained with 20 μg ml−1 propidium iodide (Sigma-Aldrich) for 10 min at room temperature. The DNA content was then determined by flow cytometry. The percentages of cells in the G0/G1, S, and G2/M phases were determined on a BD FACSCalibur (Becton Dickinson, NJ, USA), and data were analyzed with FlowJo software (Tree Star, Inc, OR, USA.).

### *In vivo* tumorigenesis assays

*In vivo* tumorigenesis and metastasis assays were performed as described previously. Briefly, 1 × 10^6^ cells were subcutaneously injected into the right flanks of nude mice. Tumor length (L) and width (W) were measured every 3 days, and tumor volume was calculated using the following equation: volume = (W2 × L)/2. After 4 weeks, the mice were sacrificed, and tumor volume and weight were measured. All animal experiments were performed with the approval of the Shandong Provincial Hospital's Animal Care and Use Committee.

### Statistical analysis

Experimental data are shown as the means ± standard deviation (s.d.). The results from different treatment groups were compared using two-tailed Student's *t*-tests. Differences were considered to be significant at *P* < 0.05. Statistical analyses were performed with SPSS/Win11.0 software (SPSS, Inc., Chicago, IL, USA).
