# Correction: Upregulation of long non-coding RNA PlncRNA-1 promotes proliferation and induces epithelial-mesenchymal transition in prostate cancer

**DOI:** 10.18632/oncotarget.27174

**Published:** 2019-08-27

**Authors:** Yang Jin, Zilian Cui, Xudong Li, Xunbo Jin, Jian Peng

**Affiliations:** ^1^ Department of Hepatobiliary Surgery, Xiangya Hospital, Central South University, Changsha, Hunan, China; ^2^ Shandong University School of Medicine, Jinan, Shandong, China; ^3^ Minimally Invasive Urology Center, Shandong Provincial Hospital affiliated to Shandong University, Jinan, Shandong, China; ^4^ Department of Urology, Binzhou People’s Hospital, Binzhou, Shandong, China

**These articles have been corrected:** The Figure 2G in ‘Upregulation of long non-coding RNA PlncRNA-1 promotes proliferation and induces epithelial-mesenchymal transition in prostate cancer,’ (https://doi.org/10.18632/oncotarget.15318), is a duplication of Figure 3F in the published article ‘PlncRNA-1 induces apoptosis through the Her-2 pathway in prostate cancer cells,’ (https://doi.org/10.4103/1008-682X.178849). The proper Figure 2G is shown below. The authors declare that these corrections do not change the results or conclusions of this paper.

Original article: Oncotarget. 2017; 8:26090–26099. 26090-26099 . https://doi.org/10.18632/oncotarget.15318

**Figure 2:**
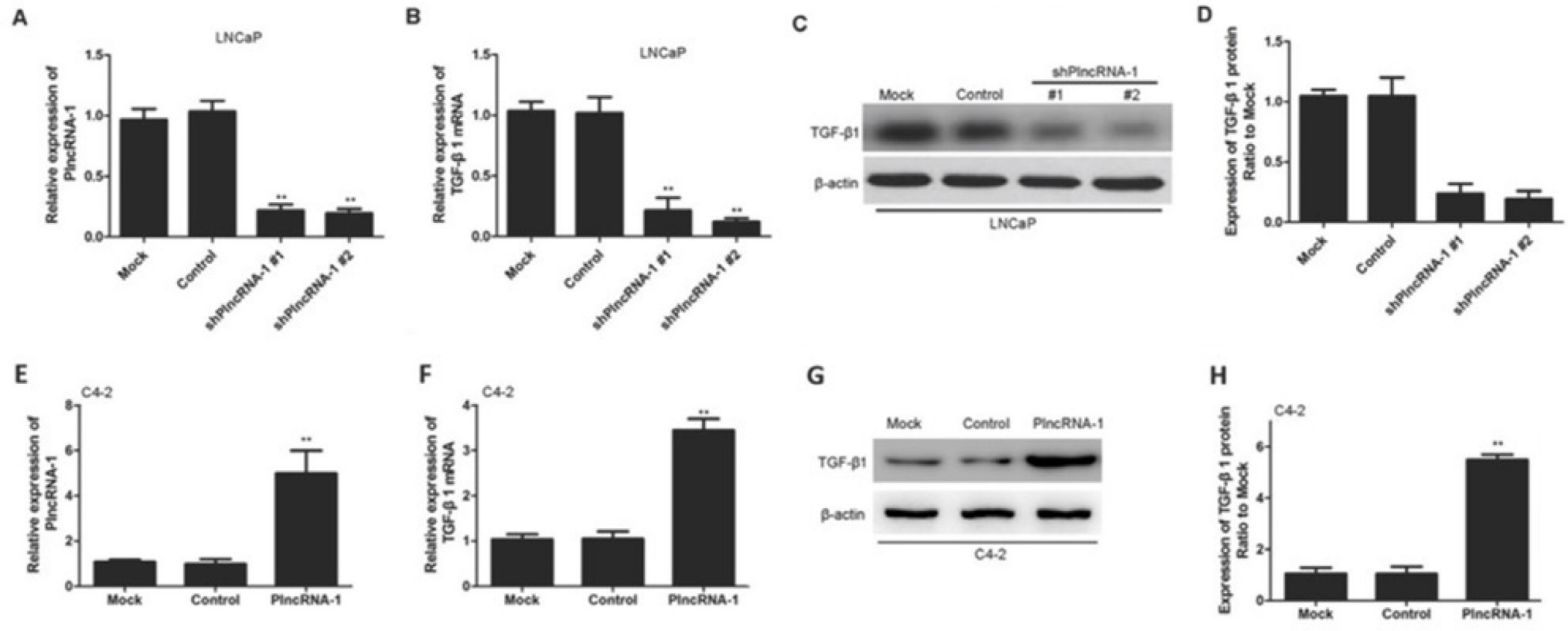
TGF-β1 expression in LNCaP and C4-2 cells after silencing and overexpression of PlncRNA-1. **A.**, **B.** Knockdown of PlncRNA-1 decreased TGF-β1 expression, as assessed by qPCR in LNCaP cells. **C.**, **D.** Knockdown of PlncRNA-1 decreased TGF-β1 expression, as assessed by Western blot in LNCaP cells. E., F. Overexpression of PlncRNA-1 upregulated TGF-β1, as assessed by qPCR in C4-2 cells. **G.**, **H.** Overexpression of PlncRNA-1 upregulated TGF-β1 expression, as assessed by Western blot in C4-2 cells.

